# Evaluation of prenatal genetic aberrations in fetuses with gallbladder abnormalities: A retrospective cohort study

**DOI:** 10.1371/journal.pone.0358236

**Published:** 2026-10-15

**Authors:** Xiaorui Xie, Xiaoqing Wu, Ying Li, Linjuan Su, Meiying Cai, Yuqin Chen, Bin Liang, Na Lin, Liangpu Xu

**Affiliations:** Medical Genetic Diagnosis and Therapy Center, Fujian Key Laboratory for Prenatal Diagnosis and Birth Defect, Fujian Provincial Maternity and Children’s Hospital, Affiliated Hospital of Fujian Medical University, Fuzhou, China; AIIMS Nagpur: All India Institute of Medical Sciences - Nagpur, INDIA

## Abstract

**Background:**

With the widespread application of chromosomal microarray analysis (CMA) in prenatal diagnosis, fetal gallbladder abnormalities have been found to be associated not only with chromosomal aneuploidies but also with certain syndromes caused by submicroscopic chromosomal structural variants. This study aimed to investigate the molecular etiology of fetal gallbladder abnormalities and to provide additional evidence for clinical screening and genetic counseling regarding these anomalies.

**Material and methods:**

This retrospective study included 258 fetuses diagnosed with gallbladder abnormalities through prenatal ultrasonographic screening. Among these, 167 cases underwent both karyotype analysis and CMA, 14 cases received CMA only, and 77 cases underwent karyotype analysis only. The fetuses were categorized into two groups based on the presence of additional structural anomalies: isolated gallbladder abnormalities (Group A), gallbladder abnormalities accompanied by soft markers (Group B) and gallbladder abnormalities accompanied by structural anomalies or other abnormalities (Group C). Within each of the three groups, cases were further subdivided into subgroups A1/B1/C1, A2/B2/C2, A3/B3/C3, and A4/B4/C4, corresponding to fetuses with nonvisualized gallbladder, gallbladder enlargement, small gallbladder, and other gallbladder abnormalities, respectively.

**Results:**

Among the total of 258 fetuses, karyotyping identified chromosomal abnormalities in 10 cases (4.2%, 10/240), whereas SNP-array detected abnormalities in 16 cases (8.7%, 16/184). The detection rate of SNP-array was higher than that of traditional chromosome karyotype analysis, and SNP-array identified submicroscopic abnormalities in 7 additional cases that were undetectable by karyotyping. The overall detection rates were significantly higher in gallbladder abnormalities accompanied by structural anomalies or other abnormalities group than that in the isolated gallbladder abnormalities group and gallbladder anomalies accompanied by soft ultrasound markers group(P < 0.05). The highest frequency of chromosomal abnormalities was observed in fetuses presenting with both gallbladder and facial abnormalities.

**Conclusion:**

For fetuses with gallbladder abnormalities, prenatal genetic diagnosis is recommended, with chromosomal microarray analysis (CMA) as the preferred testing method. It can improve the detection rate of chromosomal abnormalities associated with fetal gallbladder abnormalities and identify submicroscopic chromosomal structural variants that conventional karyotyping may miss.

## Introduction

The examination of fetal gallbladder during pregnancy has emerged as a critical component of prenatal screening and diagnosis for fetal systemic malformations. The incidence of gallbladder malformations is 0.12%−0.15%, including hypoplasia, septation, duplication, ectopia, agenesis (GBA) and gallstone [1,2]. Ultrasonography serves as the primary diagnostic modality for detecting fetal biliary system abnormalities, offering substantial clinical utility in the identification of fetal gallbladder anomalies. Although the overall incidence of fetal gallbladder abnormalities is low, their clinical significance is heightened by their potential association with chromosomal aberrations or severe conditions carrying poor prognoses, such as biliary atresia and cystic fibrosis [3]. Prenatal diagnosis of these anomalies holds considerable importance, as it enables both the prevention of severe congenital defects through informed decision-making and the optimization of postnatal management strategies.

Emerging evidence indicates that fetal gallbladder abnormalities demonstrate associations with chromosomal aneuploidies [4–9] and are implicated in several genetic syndromes, including Steinfeld syndrome, Alagille syndrome, craniomicromelic syndrome, and DiGeorge syndrome [10–13]. The widespread adoption of CMA in prenatal diagnosis has enabled the identification of additional chromosomal abnormalities undetectable by conventional karyotyping, such as microdeletions, microduplications, and regions of homozygosity (ROH) [13–15].

While previous studies have predominantly emphasized the ultrasonographic evaluation of fetal gallbladder abnormalities in prenatal diagnosis, the application of CMA in this context remains relatively unexplored. This study systematically evaluates the diagnostic utility of conventional karyotyping and single nucleotide polymorphism array (SNP-array) analysis in elucidating the genetic etiology of fetal gallbladder abnormalities during prenatal assessment.

## Materials and methods

### Study population and samples

This retrospective study enrolled 257 pregnant women who presented at Fujian Provincial Maternity and Children's Hospital in China, between January 5, 2017 and May 29, 2024, including one case of twin pregnancy (total fetuses = 258). All fetuses were prenatally diagnosed with gallbladder abnormalities through ultrasonographic screening and subsequently underwent diagnostic testing via interventional puncture procedures. The diagnosis of small gallbladder and gallbladder enlargement was based on quantitative biometric criteria using gestational age-specific reference charts for fetal gallbladder dimensions [2].Small gallbladder is defined as fetal gallbladder length and/or anteroposterior (AP) diameter measuring below the 5th percentile for the corresponding gestational week. Gallbladder enlargement is defined as fetal gallbladder length and/or AP diameter measuring above the 95th percentile for the corresponding gestational week. The following cases were excluded: (1) the cases with an uncertain diagnosis of gallbladder abnormality; (2) the cases in which genetic testing failed (e.g., cell culture failure resulting in no karyotype result); and (3) the cases where the pregnant woman refused to participate in the study or declined to provide written informed consent.

Fetuses were stratified into three cohorts based on ultrasound findings: (A) isolated gallbladder abnormalities group, (B)gallbladder anomalies accompanied by soft ultrasound markers and (C) gallbladder abnormalities accompanied by structural anomalies or other abnormalities. Within each cohort, cases were further subclassified into four categories: Group A1/B1/C1 (nonvisualized gallbladder), Group A2/B2/C2 (gallbladder enlargement), Group A3/B3/C3 (small gallbladder), and Group A4/B4/C4 (other gallbladder abnormalities).

All participants provided written informed consent prior to study inclusion. The study protocol received ethical approval from the Protection of Human Ethics Committee of Fujian Provincial Maternity and Children's Hospital.

### Chromosomal karyotyping

Fetal chromosome karyotype analysis was performed according to our laboratory's routine procedures, with resolution at the 320–400 band level. Giemsa banding (G-banding) karyotypes were analyzed and diagnosed in accordance with the International System for Human Cytogenomic Nomenclature (ISCN2020) [16].

### Chromosomal microarray analysis and data interpretation

Fetal DNA was extracted using the QIAGEN DNA mini kit (Qiagen, Valencia, CA, USA), and analyzed using the CytoScan 750K gene chip detection platform (Affymetrix Inc., CA, USA). Copy number variant (CNV) thresholds were set to report deletions greater than 200 kb or duplications greater than 500 kb. The data were analyzed using the Chromosome Analysis Suite (ChAS) software (Affymetrix, Santa Clara, CA, USA) and genomic imbalances were annotated based on the GRCh37/hg19 Genome Build (July 2013). SNP-array results were evaluated with reference to the following databases: Database of Chromosomal Imbalance and Phenotype in Humans Using Ensembl Resources (DECIPHER, http://decipher.sanger.ac.uk/), Database of Genomic Variants (DGV, http://projects.tcag.ca/variation/), Online Mendelian Inheritance in Man (OMIM, http://www.omim.org/), the ClinGen database (https://www.clinicalgenome.org/) and the ClinVar database (https://www.ncbi.nlm.nih.gov/clinvar/). Parental samples were analyzed when required for variant interpretation. CNVs were classified according to ACMG guidelines [17] into five categories: pathogenic, likely pathogenic, variants of uncertain significance (VUS), likely benign, and benign, based on integrated assessment of database records, published literature, and ultrasound findings.

### Statistical analysis

Data were expressed as frequencies and rates. Group comparisons were performed using Pearson's chi-square test or Fisher's exact test, with statistical significance set at p < 0.05.

## Results

### Clinical characteristics

The study cohort comprised 257 pregnant women with the average maternal age of 30.0 years (range: 19–44 years) and gestational ages ranging from 19 to 36 weeks. Prenatal diagnostic procedures included amniocentesis (n = 181) and cordocentesis (n = 77). Among the 258 fetuses evaluated, concurrent karyotyping and SNP-array analysis were performed in 170 cases, while 14 cases underwent SNP-array analysis alone and 74 cases received karyotyping only. Fig 1 summarizes the diagnostic workflow for fetal gallbladder abnormalities.

**Fig 1:**
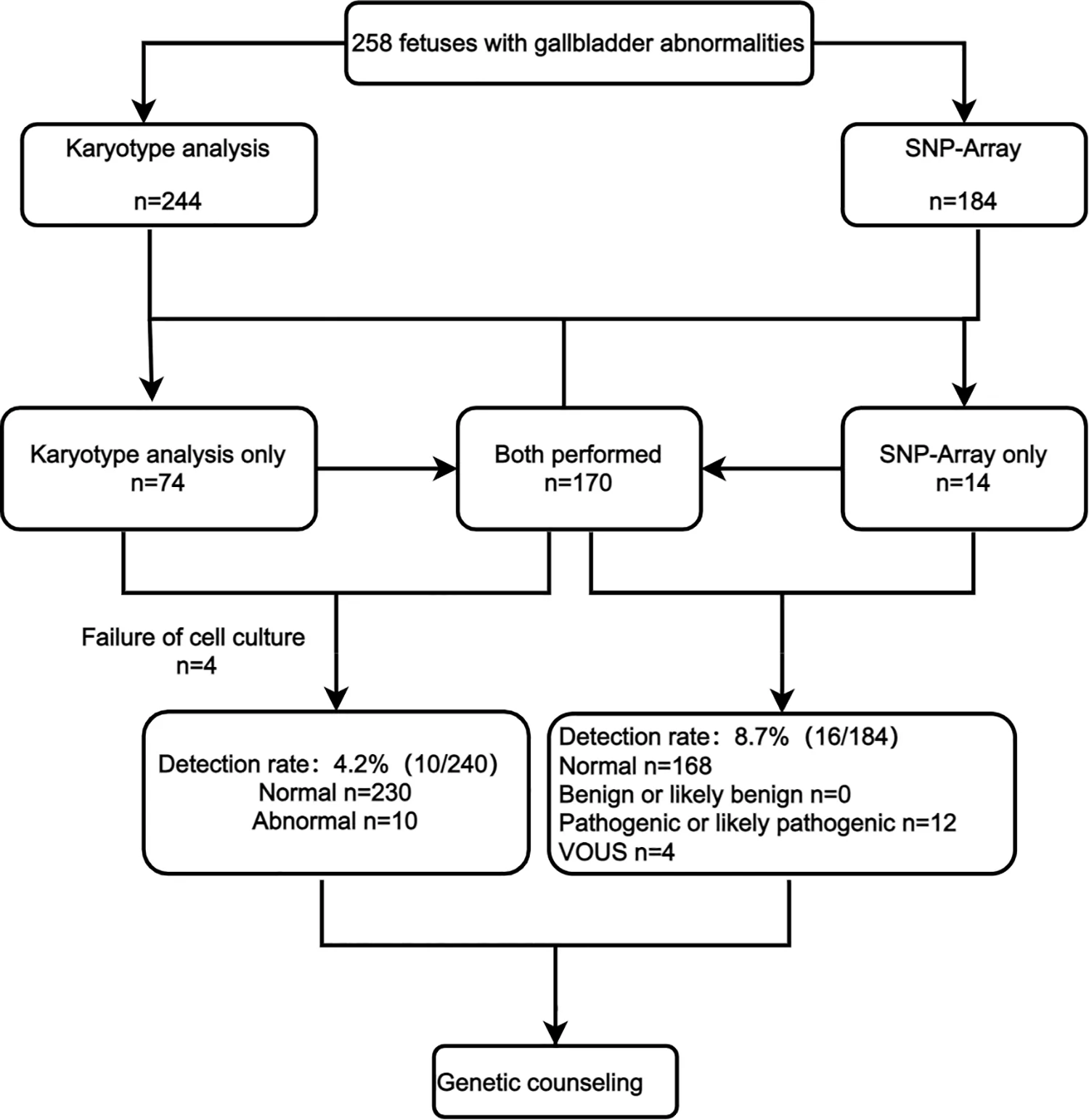
The diagnostic workflow for fetal gallbladder abnormalities.

### Results of fetal chromosome

Karyotype analysis was successfully performed in 240 of 244 attempted fetal samples (success rate: 98.4%). Among these, 230 cases (95.8%) demonstrated normal karyotypes, including six with chromosomal polymorphisms. Abnormal karyotypes were identified in 10 cases (4.2%, 10/240), with trisomy 18 being the most prevalent abnormality (n = 4). Other chromosomal abnormalities comprised trisomy 21 (n = 1), mosaicism (n = 1), and structural chromosomal aberrations (n = 4) (Table 1).

**Table 1:** Chromosomal karyotyping results.

Karyotype Classification	Karyotyping results	Number of cases
Normal	46,XX/46,XY	230
Trisomy 18	47,XX, + 18/47,XY, + 18	4
Trisomy 21	47,XY, + 21	1
Mosaicism	47,XX, + 9[29]/46,XX[26]	1
Structural chromosomal aberrations		4
	46,XX,t(2;17)(q21;q21)	1
	46,XX,del(2)(q36q36)	1
	46,XX,del(22)(q13.2)	1
	46,XY,add(16)(p13.3)	1

SNP-array analysis was successfully performed in 184 fetuses, yielding normal results in 168 cases (91.3%) and abnormal findings in 16 cases (8.7%). Although the detection rate was numerically higher than conventional karyotyping (8.7% vs 4.2%), this difference did not reach statistical significance (*χ*^*2*^ = 3.71, P > 0.05). Among the abnormal cases, six exhibited numerical chromosomal abnormalities (including one mosaicism), while ten demonstrated CNVs. Notably, SNP-array identified seven cases of submicroscopic abnormalities undetectable by karyotyping, excluding three cases with large-segment structural abnormalities. In one such case, SNP-array additionally revealed a clinically significant microdeletion. Pathogenic or likely pathogenic CNVs were identified in six cases, including Phelan-McDermid syndrome and DiGeorge syndrome (DGS), with the remaining four classified as VUS. Comprehensive characteristics of all CNV cases are detailed in Table 2.

**Table 2:** Genomic and ultrasonographic characteristics of 10 fetuses with CNVs.

Cases	GA (weeks)	Type of specimen	SNP result	Size	Ultrasound findings	Inheritance pattern *	Clinical significance	Pregnancy outcomes
Variants of pathogenic or likely pathogenic								
1	24+	Amniotic fluid	2q36.1q36.3(223547424-230792924)x1	7.2Mb	Small gallbladder + FGR, LVEF, reduced TCD	*De novo*	Pathogenic	TOP
2	27+	Amniotic fluid	15q11.2(22770421-23277436)×1	507Kb	Small gallbladder + mild TR	Maternal	pathogenic	Live birth
3	29+	Cord blood	16p13.3(85880-536631)×1; 17q24.2q25.3(64966574-81041823)×3	451Kb; 16Mb	Enlarged gallbladder + FGR, VSD, umbilical vein internal segment was widened, mild TR, ventriculomegaly, subependymal cyst	*De novo*	Pathogenic; Pathogenic	TOP
4	24+	Amniotic fluid	17p12(14099565-15424086)×3	1.3Mb	Small gallbladder + mild TR; BPD, HC, AC were below the normal predicted value	*De novo*	Pathogenic	Live birth
5	33+	Amniotic fluid	22q11.21(18631365-21800471)×1	3.1Mb	Nonvisualized gallbladder + larger stomach bubble	*De novo*	Pathogenic	TOP
6	26+	Amniotic fluid	22q13.2q13.33(43784093-51197766)×1	7.4Mb	Nonvisualized gallbladder + reduced TCD	*De novo*	Pathogenic	TOP
Variants of unknown significance								
7	25+	Amniotic fluid	4p15.2(24623766-25367018)×1	743Kb	Small gallbladder	Paternal	VOUS	Live birth
8	29+	Amniotic fluid	6q26(162562481-163102082)×1	540Kb	Enlarged gallbladder + hydramnios	*De novo*	VOUS	Lost to follow-up
9	32+	Amniotic fluid	8p22(13599177-16450454)×1	2.8Mb	Nonvisualized gallbladder + mild TR, cardiothoracic ratio increased	Paternal	VOUS	Live birth
10	25+	Amniotic fluid	16p13.13p13.12(11528493-12934811)×3	1.4Mb	Enlarged gallbladder + Nasal bone hypoplasia	Unknown	VOUS	Live birth

* Unknown: The patient refused to undergo pedigree verification.

GA: gestational ages; FGR: fetal growth restriction; LVEF: left ventricular echogenic foci; TCD: transverse cerebellar diameter; TR: tricuspid regurgitation; TOP: termination of pregnancy; VSD: ventricular septal defect; BPD: biparietal diameter; HC: head circumference; AC: abdomen circumference

### Distribution of chromosomal abnormalities in different groups

The cohort comprised 258 cases categorized into three groups: Group A with 53 cases (20.5%, 53/258), Group B with 93 cases (36.1%, 93/258), and Group C with 112 cases (43.4%, 112/258). Karyotype analysis demonstrated detection rates of 0% (0/51) in group A, 0% (0/87) in group B and 9.8% (10/102) in group C. Corresponding SNP-array detection rates were 2.7% (1/37), 2.9% (2/69), and 16.7% (13/78), respectively. Although both methods showed differences were statistically significant among the three groups (karyotype: P = 0.0060 < 0.05, SNP-array: P = 0.0024 < 0.05). After pairwise comparisons, it was found that group C had a significantly higher rate than group A and B [karyotype: P = 0.0312 < 0.05 (A vs. C), P = 0.0020 < 0.05 (B vs. C); SNP-array: P = 0.0227 < 0.05 (A vs. C), P = 0.0029 < 0.05 (B vs. C)].

According to the different manifestations of gallbladder abnormalities, cases were further subclassified into four categories. 74 cases were of nonvisualized gallbladder, 129 were of gallbladder enlargement, 48 were of small gallbladder and 7 cases had other gallbladder abnormalities. Focusing on the SNP‑array results with relatively higher detection rates, the chromosome abnormality detection rates in groups A1, A2, A3, and A4 were 0%, 0%, 9.1%, and 0%, respectively; in groups B1, B2, B3, and B4, they were 0%, 3.1%, 5.3%, and 0%, respectively; and in groups C1, C2, C3, and C4, they were 19.2%, 23.2%, 13.1%, and 0%, respectively. Fetuses with small gallbladder demonstrated the highest frequency of chromosomal abnormalities across the three groups. However, pairwise comparisons among the four phenotypic groups revealed no statistically significant differences in detection rates (P > 0.05). The details are shown in Table 3.

**Table 3:** Detection rates of chromosomal abnormalities by phenotypic group.

Phenotypic group	Karyotyping			SNP-array			
	Abnormal	Total	Detection rate (%)	Abnormal	Total	Detection rate (%)	P
Isolated gallbladder abnormalities group							
Group A1	0	16	0%	0	15	0%	
Group A2	0	22	0%	0	10	0%	
Group A3	0	12	0%	1	11	9.1%	
Group A4	0	1	0%	0	1	0%	
Total	0	51	0%	1	37	2.7%	0.5950^*^
Gallbladder abnormalities accompanied by soft markers							
Group B1	0	19	0%	0	17	0%	
Group B2	0	47	0%	1	32	3.1%	
Group B3	0	19	0%	1	19	5.3%	
Group B4	0	2	0%	0	1	0%	
Total	0	87	0%	2	69	2.9%	>0.9999^*^
Gallbladder abnormalities accompanied by structural anomalies or other abnormalities							
Group C1	4	30	13.3%	5	26	19.2%	
Group C2	4	55	7.3%	5	38	13.2%	
Group C3	2	14	14.3%	3	13	23.1%	
Group C4	0	3	0%	0	1	0%	
Total	10	102	9.8%	13	78	16.7%	0.7230 ^*^

*The P-values were calculated for the overall comparison across subgroups within each group. They were not applicable to individual subgroups.

Among fetuses with gallbladder abnormalities plus extra ultrasound anomalies, the highest chromosomal abnormality rates occurred in those with gallbladder abnormalities plus facial anomalies (karyotype: 50%; SNP-array: 50%), followed by nervous system anomalies (karyotype: 21.4%; SNP-array: 33.3%). The complete distribution across anomaly types is presented in Table 4.

**Table 4:** Gallbladder abnormalities with concurrent ultrasound findings in other systems.

System involved	Analyzed cases^†^			Abnormal cases (%)	
	Total	Karyotype	SNP-array	Karyotype	SNP-array
Cardiovascular system	125	117	96	9(7.7)	11(11.5)
Urinary system	39	35	27	2(8.3)	2(7.4)
Nervous system	29	28	18	6(21.4)	6(33.3)
Other digestive system	38	34	27	2(5.9)	3(11.1)
Skeletal abnormalities	9	8	7	1(12.5)	2(28.6)
Facial abnormalities	5	4	4	2(50.0)	2(50.0)
limb abnormalities	6	6	4	1(16.7)	1(25.0)
Other findings^‡^	58	54	36	5(9.3)	7(19.4)

^†^ Cases with multiple system involvement were counted in each relevant category.

^‡^ Includes: polyhydramnios, oligohydramnios, increased NT or NF thickness, fetal growth restriction (FGR), intrauterine growth restriction (IUGR), single umbilical artery (SUA), etc.

### Pregnancy outcomes

Among the 258 fetuses included in this study, pregnancy outcome data were available for 232 cases (follow-up rate: 89.9%). Elective pregnancy termination occurred in 15 cases (6.5%), including one trisomy 21, four trisomy 18 and one pathogenic CNV. Among the 212(91.4%) cases of live births, 202 cases were live birth with normal phenotype, including 198 cases with normal chromosomal results, one with balanced translocation, and three with VOUS CNVs. Complete pregnancy outcomes dates were presented in Table 5.

**Table 5:** Pregnancy outcomes of the total cohort.

Pregnancy outcomes	Cases with normal chromosomes	Cases with chromosomal aberrations				
		Trisomy 21	Trisomy 18	Balanced translocation	pathogenic/likely pathogenic CNVs	VUS
Live birth with normal phenotype	198^a^	0	0	1^d^	2	3
Live birth with abnormal phenotype	10^b^	0	0	0	0	0
Termination of pregnancy	9^c^	1	4	0	4	0
Lost to follow-up	24	0	0	0	0	1
Total	241	1	4	1	6	4

a: Includes 6 preterm deliveries and 1 neonatal death (day 13)

b: Abnormal phenotypes comprised: 5 neonatal jaundice cases (1 with gastrointestinal bleeding, 1 with gallbladder enlargement and FGR), 1congenital cystic duct cyst, 1 gallbladder enlargement with cryptorchidism, 1 ventricular septal defect with patent ductus arteriosus, 1 neonatal patent foramen ovale, and 1 atrial septal defect.

c: 8 abortions for severe malformation, 1 stillborn

d: Balanced translocation: 46,XX,t(2;17)(q21q21).

## Discussion

Existing literature has documented chromosomal abnormalities in fetuses with gallbladder abnormalities include chromosomal aneuploidy, such as trisomy 13 [18,19], trisomy 18 [8,19], trisomy 22 [5], and trisomy 16 [20]. Chromosomal copy number variants were also detected, such as DiGeorge syndrome (22q11.2 microdeletions) [8,13,21], 8p duplication [22], 14q32.1 microduplication [23], 16q duplication [24]. In this study, the detection rate of chromosomal abnormalities was 4.2% by karyotype analysis and 8.7% by SNP-array, with trisomy 18 being the most prevalent abnormality. Consistent with prior reports [8,19], all trisomy 18 cases in our cohort were combined with extra ultrasound anomalies such as cardiac malformations and fetal growth restriction(FGR). The results of fetal chromosome showed that the detection rate by SNP-array appeared higher than traditional chromosome karyotype analysis. This difference was not statistically significant(P > 0.05), however, SNP-array identified seven cases of submicroscopic abnormalities undetectable by karyotyping, including both pathogenic or likely pathogenic variants. Case 2 in Table 2 showed a 507Kb maternally inherited microdeletion at 15q11.2. The region contains 4 OMIM genes, including *TUBGCP5* (608147), *CYFIP1* (606322), *NIPA2* (608146) and *NIPA1* (608145). This pathogenic variant demonstrates variable expressivity, ranging from normal phenotypes to clinical manifestations including developmental delay, epilepsy, autism spectrum disorder (ASD), and congenital heart disease (CHD) [25,26]. Case 3 had a large fragment of duplication in chromosome 17 combined with a microdeletion in 16p13.3 undetected by karyotype analysis. The region of duplication in chromosome 17 was 16.0Mb in size and contains 160 OMIM genes, associated with congenital anomalies including developmental delay, intellectual disability, epilepsy and microcephaly. The region of microdeletion in 16p13.3 was 451Kb in size and contained 17 OMIM genes. It is also a pathogenic variant which is associated with alpha-thalassemia/mental retardation linked to chromosome16(ATR-16) syndrome. Case 4 demonstrated a 1.3Mb microduplication in the region of 17p12. It contains 4 OMIM genes, including *PMP22*(601097) known to cause Charcot-Marie-Tooth type 1A(CMT1A). This neuropathy is characterized by progressive distal muscle weakness and atrophy(predominantly affecting hands and lower limbs), hearing impairment, pes cavus, and hip dysplasia. Case 5 carried a 3.1Mb microdeletion in 22q11.21. which is associated with DiGeorge syndrome, manifesting with developmental delay, cardiac defects, thymic hypoplasia, characteristic facies, and neurodevelopmental abnormalities. These findings underscore the clinical necessity of CMA when an ultrasound suggests fetal gallbladder abnormalities detected ultrasonographically, particularly in cases with normal karyotyping.

Among the four VUS cases, three underwent family verification: one was a *de novo* variant (case 8), and two were inherited from phenotypically normal fathers (cases 7 and 9). The remaining case (case 10) did not undergo verification because the parents declined. However, inheritance from a phenotypically normal parent does not necessarily indicate a benign variant. The 4p15.2 deletion in case 7 involves three OMIM genes, among which the homozygous mutations of the *TUSC3* (601385) gene are associated with autosomal recessive intellectual disability type 7. The 8p22 deletion in case 9 involves the *SEPSECS* gene, mutations of which are linked to pontocerebellar hypoplasia type 2D (PCH2D) [27,28]. For the VUS cases, in the prenatal consultation, a multidisciplinary team including genetic counselors, obstetricians, and neonatologists provided personalized consultations to parents. VUS were explained as gene variation with currently unclear clinical significance, neither confirming nor excluding a pathological condition. And follow-up ultrasound examinations were recommended to monitor changes in structural abnormalities.

In our study, chromosomal abnormalities were predominantly identified in fetuses with abnormal gallbladder accompanied by structural anomalies or other abnormalities (Group A). The detection rate of chromosome abnormalities in Group A was significantly higher than that in Groups B and C, which was similar to previous research findings [8,15]. However, it cannot definitively exclude the potential association between isolated gallbladder abnormalities and gallbladder abnormalities accompanied by soft markers and the risk of chromosomal abnormalities. In Groups B and C, one pathogenic variant (case 2) and two variants of uncertain significance (case 7 and 10) were detected. Consequently, clinical decision-making regarding prenatal diagnostic testing should incorporate comprehensive evaluation of serial ultrasound findings, family history, and ancillary diagnostic results.

Based on the ultrasonographic manifestations of gallbladder abnormalities, the cases were categorized into four groups: nonvisualized gallbladder, gallbladder enlargement, small gallbladder and other gallbladder abnormalities. Nonvisualized gallbladder represents a rare clinical finding, with a reported incidence of 0.1%−0.15% in prenatal examinations [4,23,29]. The underlying etiologies are multifactorial, encompassing gallbladder hypoplasia, chromosomal aberrations, cystic fibrosis, and extrahepatic biliary atresia [1,3,4,30]. It has been reported that isolated gallbladder enlargement may be a normal variation. In our cohort, no chromosomal abnormalities were detected in cases with isolated gallbladder enlargement, which is consistent with the previous research results [19]. Limited data exist evaluating and reporting on a smaller gallbladder. Other gallbladder abnormalities, particularly choledochal cysts, demonstrate even lower prevalence rates. In this study, only one case of congenital choledochal cysts was identified, which showed no associated chromosomal abnormalities. This case underwent successful postnatal surgical intervention with favorable outcomes. Notably, our analysis revealed no statistically significant differences in chromosomal abnormality detection rates across various gallbladder abnormality subtypes, irrespective of concomitant other ultrasound findings (P > 0.05). Among fetuses with gallbladder abnormalities accompanied by other manifestations of ultrasound abnormalities, the highest frequency of chromosomal aberrations occurred in cases with concurrent facial abnormalities, followed by those with nervous system anomalies. The chromosomal abnormalities identified in fetuses with concurrent gallbladder and facial abnormalities comprised trisomy 21 and trisomy 18. Both conditions are characterized by multi-system developmental delays, including the digestive system and distinctive craniofacial features. We identified one case with concomitant gallbladder and neurological abnormalities carried a deletion in 22q13.2q13.33 which contains 55 OMIM genes including *MPPED1* (602112), *SULT4A1* (608359), *PNPLA5* (611589), *PNPLA3* (609567), *SHANK3* (606230) and *ACR* (102480). This genomic region is associated with PM syndrome, which typically presents with global developmental delay, moderate to severe intellectual disability and neurological manifestations include autistic behaviors, neonatal hypotonia. It has also been reported that some children have potential gastrointestinal involvement such as gastroesophageal reflux.

Analysis of pregnancy outcomes (excluding cases lost to follow-up) revealed that 11.5% (25/217) of fetuses with normal chromosomal results exhibited clinical phenotypes postnatally, among which there were 8 cases involving the gallbladder or digestive system manifestations. The remaining 88.5% (192/217) showed no apparent abnormalities, potentially attributable to false-positive ultrasound findings resulting from physiological gallbladder contraction [31,32]. However, early detection and diagnosis remain clinically imperative, as fetal gallbladder abnormalities may indicate serious conditions including biliary atresia, gallbladder hypoplasia, and cystic fibrosis. Importantly, fetuses with normal chromosomes still present with related malformations necessitating early intervention.

## Conclusions

CMA demonstrates superior diagnostic utility in prenatal evaluation of gallbladder abnormalities, enhancing the detection rate of chromosomal aberrations and enabling identification of submicroscopic structural variants. Our findings support CMA as the primary diagnostic modality for such cases. The chromosomal abnormality rates were higher in fetuses with structural anomalies or other abnormalities, with statistical significance. Nevertheless, isolated gallbladder anomalies cannot be ignored, necessitating comprehensive clinical assessment. Early prenatal detection and appropriate intervention for gallbladder abnormalities carry substantial clinical importance, facilitating optimal pregnancy management and enabling timely postnatal therapeutic interventions when indicated.
